# Microglia-derived extracellular vesicle content as a biomarker for early detection of Alzheimer’s disease

**DOI:** 10.1186/s40035-026-00587-9

**Published:** 2026-09-30

**Authors:** Amber L. Woudstra, Linda Holtman, Erwin A. van Vliet

**Affiliations:** 1 Leiden Academic Centre for Drug Research, Leiden University, Leiden, Netherlands; 2 Department of Biomedical Sciences, University of Groningen, University Medical Center Groningen, Groningen, the Netherlands; 3 Swammerdam Institute for Life Sciences, Center for Neuroscience, University of Amsterdam, Science Park 904, 1098 XH Amsterdam, the Netherlands; 4 Department of (Neuro) Pathology, Amsterdam Neuroscience, Amsterdam UMC Location University of Amsterdam, Meibergdreef 9, Amsterdam, the Netherlands

**Keywords:** Early diagnosis, Disease-associated microglia, Dementia, Measurability, Sensitivity, Specificity

## Abstract

Alzheimer’s disease (AD) is a progressive neurodegenerative disorder characterized by amyloid-beta aggregation, tau pathology, synaptic dysfunction, and neuronal loss. While current biomarker frameworks such as the A/T/N classification have advanced diagnosis, they predominantly reflect relatively late stages of disease, limiting opportunities for early intervention. This underscores the urgent need for biomarkers that are not only accessible and sensitive but also specific to the earliest pathogenic mechanisms of AD. Recent work has underscored the pivotal role of disease-associated microglia (DAM) in the onset and progression of AD. DAM exhibit dual roles: they promote protection by phagocytosing amyloid and debris, metabolizing lipids, and remodelling synapses, yet can also drive pathology through excessive cytokine release, chronic inflammatory signalling, and impaired aggregate clearance. DAM release extracellular vesicles (EVs) that encapsulate proteins, nucleic acids, and lipids, reflective of their state. These vesicles reflect microglial (dys)function and can be detected in cerebrospinal fluid and blood, offering a minimally invasive means to monitor early AD pathology. In this review, we examine the emerging evidence that the content of microglia-derived EVs may fulfil the criteria of ideal biomarkers with a focus on the detectability, sensitivity, specificity, and feasibility for longitudinal monitoring. We discuss their potential to overcome limitations of current fluid and imaging biomarkers by offering more stable cargo protection, cell-type specificity, and accessibility in peripheral fluids. Furthermore, we highlight the opportunities and challenges in isolating microglia-derived EVs, identifying reliable surface and cargo markers, and standardizing methodologies for reproducibility across studies. By integrating insights from microglial biology with EV research, we provide evidence for microglia-derived EV content as biomarkers for early detection of AD and propose a roadmap toward DAM-specific EV signatures that could transform early AD diagnosis and disease monitoring. Such advances hold promise not only for AD but also for broader applications in neurodegenerative and neuroinflammatory disorders.

## Background: current validated biomarkers for Alzheimer’s disease (AD)

AD is a progressive neurodegenerative disorder characterized by the accumulation of amyloid-beta (Aβ), hyperphosphorylated tau, gliosis (including microgliosis and astrogliosis), neuroinflammation, and synaptic dysfunction, ultimately leading to neurodegeneration and cognitive as well as functional decline. Early diagnosis of AD is critical for intervention at the earliest possible stages utilizing biomarkers to identify and monitor the progression of the disease effectively.

Established AD-specific biomarkers include indicators of amyloid and tau pathology, synaptic and neuronal loss, and neuroinflammation [1]. These can be assessed in cerebrospinal fluid (CSF), blood, or in situ using imaging techniques such as positron emission tomography (PET) and magnetic resonance imaging. Since 2018, the A/T/N biomarker-based classification system has been applied clinically to integrate biomarker findings [2]. This framework includes: (A) Aβ, assessed as Aβ42 in CSF or by amyloid-PET imaging; (T) tau, measured as total or phosphorylated tau in CSF or by tau-PET imaging; and (N) neurodegeneration, evaluated through structural imaging of brain atrophy or neurodegeneration markers such as neurofilament light chain. Current fluid and imaging biomarkers within the A/T/N framework offer high diagnostic accuracy for established AD pathology. Among these, plasma p-tau217 level and mass-spectrometry-based Aβ42/40 ratio now show sensitivity and specificity that are comparable to those of PET and CSF assays [3, 4]. However, these markers primarily reflect downstream neuronal and proteinopathic processes and may be less sensitive to the initiating microglial or neuroimmune events that precede them [2, 5], and protein levels may be altered outside the brain. Blood samples may also be confounded by non-neuronal proteins, such as peripheral tau [6]. Some biomarkers lack disease specificity; for example, neurofilament light chain reflects general neurodegeneration [7], while α-synuclein (a key component of Lewy bodies) is present in only about half of AD cases [8]. Another challenge lies in distinguishing AD from non-AD dementias at the mild cognitive impairment (MCI) stage [1, 9].

**Fig. 1:**
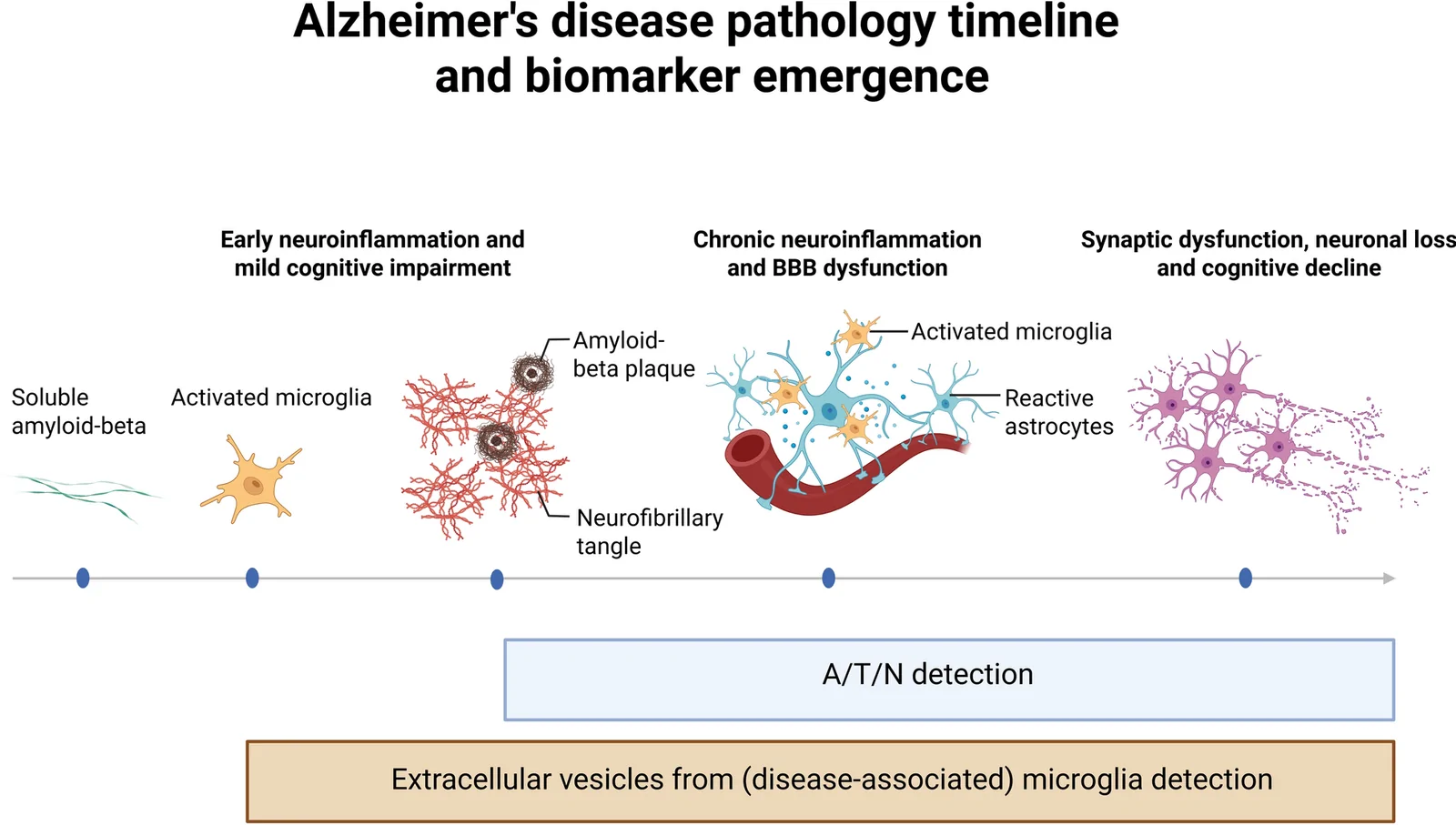
Alzheimer’s disease (AD) pathology timeline and biomarker emergence. The schematic timeline of AD progression shows sequential pathological events: increase in soluble amyloid-beta, early neuroinflammation with activated microglia and mild cognitive impairment, amyloid-beta deposition (plaques) and tau hyperphosphorylation (neurofibrillary tangles), chronic neuroinflammation with activated microglia and astrocytes and blood–brain barrier (BBB) dysfunction, synaptic dysfunction, neuronal loss, and cognitive decline. The detectability of classical A/T/N biomarkers (amyloid, tau, neurodegeneration markers) is compared with that of extracellular vesicle biomarkers from (disease-associated) microglia, which appears earlier and enables prodromal detection of AD. Created in https://BioRender.com

These hurdles underscore the need for new strategies to identify early, disease-specific biomarkers for AD. In this review, we focus on extracellular vesicles (EVs) from microglia that contain proteins, lipids, and nucleic acids as early and accessible fluid biomarkers for AD, reflecting microglial activation and could complement the existing A/T/N biomarkers (Fig. 1). EV-based tests have entered clinical practice in other fields. For example, the urine exosome gene-expression assay ExoDx Prostate (IntelliScore) is used to stratify risk of high-grade prostate cancer in men with PSA (prostate-specific antigen) 2–10 ng/mL [10, 11]. The ExoDx Prostate test is offered as a Clinical Laboratory Improvement Amendments-certified laboratory-developed test and has received FDA Breakthrough Device designation, with reimbursement by Medicare and several commercial insurers; however, it has not received full FDA premarket approval or clearance [12]. EV-based assays for bladder and lung cancer are currently limited to research-use or early-stage clinical evaluation, without regulatory approval for routine care [13]. As of 2026, there are no FDA-approved EV-based biomarkers for routine AD diagnosis. This contrast highlights that, even in oncology where liquid biopsies are relatively advanced, translating EV-based biomarkers into widely approved diagnostic tools remains challenging—a hurdle that is even more pronounced in AD. However, as novel diagnostic biomarkers for neurological diseases, EVs demonstrate remarkable sensitivity and specificity in the early diagnosis, disease monitoring, and prognostic evaluation of conditions such as Parkinson’s disease and stroke [13]. Similarly, promising results are obtained for AD [14].

## Microglia activation during the early phase of AD

Aβ plaques and tau hyperphosphorylation are well-established neuropathological hallmarks of AD. However, growing evidence suggests that neuroinflammation and microglial activation may occur even earlier in the disease process [15, 16]. Initially, microglial activation plays a protective role by clearing misfolded proteins and damaged synapses. Once the burden of pathological stressors exceeds a critical threshold, however, microglia lose their ability to maintain central nervous system (CNS) homeostasis. In this dysfunctional state, they can amplify neuroinflammation instead of resolving it, as has been shown in animal models [17, 18].

Based on converging evidence from experimental and clinical studies, dysfunctional microglia in AD and other neurodegenerative conditions are widely proposed to contribute to disease progression through impaired clearance of Aβ [19] and tau aggregates [20] and through exaggerated inflammatory responses with excessive cytokine release, thereby promoting chronic neuroinflammation and neuronal damage [17, 21]. Once initiated, neuroinflammation may become self-perpetuating, as activated microglia recruit neighbouring microglia and astrocytes [21, 22], establishing chronic CNS inflammation that accelerates AD progression [17, 22].

Given their central role, microglia represent a promising source of biomarkers in the early phases of AD. However, currently, there is a lack of specific and sensitive microglial biomarkers which can accurately measure preclinical AD. Recently, the focus has shifted towards biomarkers reflecting microglial states that could serve as indicators of early neuroinflammatory processes, enabling closer monitoring of disease progression and supporting timely interventions. A particularly important development in understanding microglial contributions to AD has been the identification of disease-associated microglia (DAM), which exhibit distinct molecular and functional characteristics compared to homeostatic microglia.

### DAM

DAM have been identified using single-cell RNA sequencing (scRNA-seq) and single-nuclei RNA sequencing (snRNA-seq) in mouse models of AD. They display distinct transcriptional, secretory, and functional profiles compared to homeostatic microglia [23–25]. Importantly, DAM represent one of several microglial activation states; interferon‑responsive, major histocompatibility complex (MHC) class II–high, lipid‑droplet-accumulating microglia (LDAM), and other subpopulations have also been described in neurodegenerative models and during aging, and likely contribute to the overall microglial response in AD [25, 26]. DAM upregulate genes linked to immune responses and phagocytosis, while downregulating genes that maintain homeostasis, resulting in a dysregulated and pathological phenotype, contributing to excessive neuroinflammation and neurotoxicity [23, 27]. In current usage, “DAM” primarily denotes a specific transcriptomic module rather than a discrete, homogeneous functional cell type, and DAM-like signatures are observed in only a subset of microglia within affected brain regions in mouse models [23, 24]. This implies that changes in EV or exosomal composition during disease onset may reflect the combined output of multiple co‑existing microglial states, not DAM alone.

DAM are primarily activated by NAMPs (neurodegeneration-associated molecular patterns)—conserved structural molecular features such as aggregated or misfolded proteins, altered lipids, and nucleic acids that signal neuronal injury—which are closely related to damage-associated molecular patterns, host-derived danger molecules released during cellular stress that distinguish them from foreign, microorganism-derived PAMPs (pathogen-associated molecular patterns) [28], which position DAM as key responders to neurodegeneration in the CNS. DAM exhibit dual roles in AD: they promote protection by phagocytosing amyloid and debris, metabolizing lipids, and remodelling synapses, yet can also drive pathology through excessive cytokine release, chronic inflammatory signalling, and impaired aggregate clearance [29]. However, it is important to note that the DAM concept was originally defined in transgenic AD mouse models, which only partially recapitulate the complexity, co-pathologies, and temporal course of human AD, limiting the direct translational relevance of murine DAM signatures to human disease [30].

**Fig. 2:**
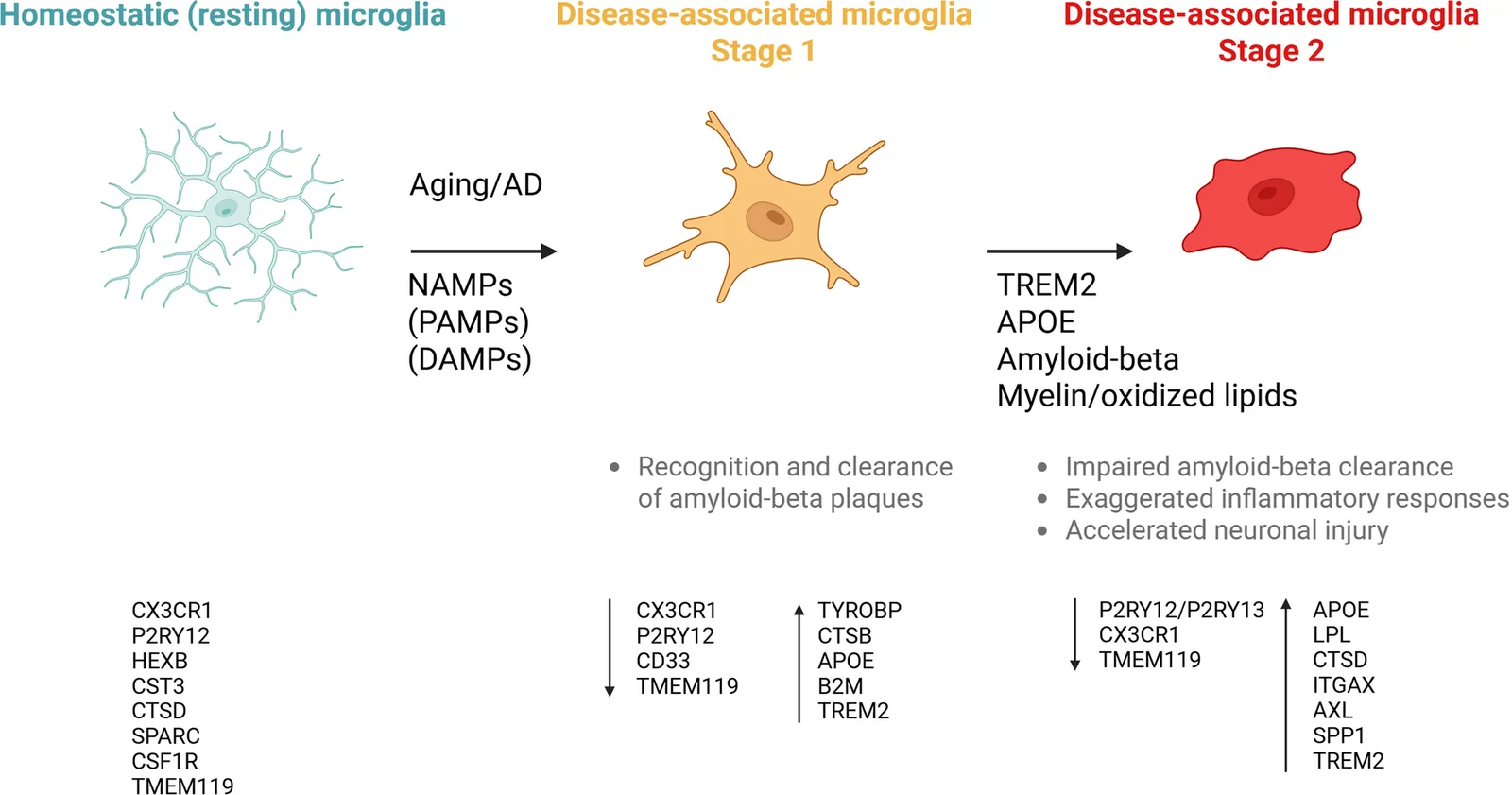
Activation cascade of disease-associated microglia. Homeostatic (resting) microglia are primarily activated by neurodegeneration-associated molecular patterns (NAMPs), which transform these resting microglia into stage 1 DAM, resulting in changes in expression of homeostatic genes, such as *CX3CR1* and *P2RY1*2. Pathogen-associated molecular patterns (PAMPs) and damage-associated molecular patterns (DAMPs) may also contribute, but they typically steer microglia toward a transient pro-inflammatory state rather than the DAM stage. DAM exhibit dual roles: they promote protection by phagocytosing amyloid and debris, metabolizing lipids, and remodelling synapses, yet can also drive pathology through excessive cytokine release, chronic inflammatory signalling, and impaired aggregate clearance. During AD progression, DAM transition from ‘stage 1’ to ‘stage 2’, which is TREM2-dependent, resulting in changes in gene expression, including *APOE*, *CTSD*, and *LPL*. Arrows indicate genes that are up- or downregulated at each stage. Created in https://BioRender.com

During AD progression, DAM transition from ‘stage 1’ to ‘stage 2’ [23]. This transition is tightly regulated by triggering receptor expressed on myeloid cells 2 (TREM2), a transmembrane receptor critical for microglial activation, proliferation, phagocytosis, and cytoskeletal remodelling (Fig. 2) [23]. Mutations in *TREM2* are known AD risk factors associated with greater plaque accumulation and neuronal loss [31]. Stage 1 DAM are activated independently of TREM2, resulting in downregulation of homeostatic genes, such as *CX3CR1* and *P2RY1*2 [23]. Transition to stage 2 DAM requires TREM2-dependent signalling, initiated by binding of lipid and apolipoprotein ligands (e.g., ApoE-bound Aβ and myelin/oxidized lipids) to TREM2 on microglia. This engagement signals via the adaptor TYROBP (also known as DAP12) and downstream kinase cascades, driving transcriptional reprogramming characterized by increased phagocytic activity and upregulation of lipid metabolism genes, including *APOE*, *CTSD*, and *LPL* [28].

TREM2 recognizes a range of lipid and damage-associated ligands, including apolipoproteins and amyloid-related lipoprotein complexes, and signals via TYROBP (DAP12) to regulate microglial survival, chemotaxis, phagocytosis and lipid handling [32, 33]. Rare coding variants in *TREM2*, such as R47H and other missense changes, confer a substantially increased risk of late-onset AD and are generally considered hypomorphic alleles that attenuate TREM2 signalling and impair the full transition to the DAM state [32]. *APOE*, in turn, is the strongest common genetic risk locus for sporadic AD; the *APOE* ε4 allele increases AD risk in a dose-dependent manner and is associated with altered Aβ aggregation and clearance, as well as with a shift of microglia toward a more inflammatory, less homeostatic phenotype [34]. Transcriptomic studies have identified a TREM2–APOE axis in which TREM2‑dependent upregulation of *APOE* and other lipid metabolism genes, including *CTSD* and *LPL*, accompanies the switch from homeostatic microglia to DAM [23, 27]. In this context, *APOE* genotype and *TREM2* loss‑ or gain‑of‑function variants modulate the emergence, intensity and quality of the DAM response, and are likely to influence the molecular composition of microglia-derived EVs, particularly their lipid and protein cargoes, in AD.

Insights from the 5×FAD mouse model of AD further illustrate DAM dynamics. Single-cell RNA-seq in whole brains of 5×FAD mice was performed at 1, 3, 6, and 8 months of age [23]. In order to define the temporal transcriptional changes leading to DAM formation, a mathematical model was generated based on a generalized logistic function to describe the temporal changes in gene expression. This model depicts the molecular order of events in which microglia are switching from the homeostatic state to DAM. At 3 months, corresponding to early plaque deposition (but well before neuronal loss and cognitive dysfunction are evident), microglia begin to acquire an ‘early’ or stage 1 DAM signature, characterized by partial downregulation of homeostatic genes (e.g. *P2ry12, Tmem119, Cx3cr1*) and upregulation of genes involved in phagocytosis and lipid metabolism (e.g. *Apoe, Lpl, Tyrobp*), which is largely TREM2-independent [23]. Stage 2 DAM emerge by 6 months, coinciding with more advanced amyloid pathology and the onset of behavioural and cognitive deficits in 5×FAD mice, indicative of symptomatic disease progression in this model, with robust induction of genes such as *Trem2, Axl, Cst7, Itgax* and *Clec7a* and a more complete suppression of the homeostatic microglial signature [23].

Similar transcriptional changes have been described for a Microglial Neurodegenerative phenotype (MGnD) in multiple mouse models of neurodegenerative disease, including AD, amyotrophic lateral sclerosis, and prion disease. Krasemann et al. [27] identified a TREM2–APOE-driven MGnD program characterized by downregulation of homeostatic microglial genes such as *P2ry12, Tmem119* and *Cx3cr1* and upregulation of genes including *Apoe, Axl, Lpl, Cst7* and *Itgax*. This MGnD signature shows substantial overlap with the DAM transcriptional profile described in amyloid mouse models, suggesting that DAM and MGnD represent related neurodegeneration-associated microglial states along a common TREM2–APOE axis, observed across distinct neurodegenerative contexts.

## Identifying AD-specific microglial responses

Although DAM are not unique to AD [23], certain markers and transcriptional profiles are particularly enriched in AD, suggesting a degree of disease specificity. Similar DAM-like subpopulations have been described in other neurodegenerative diseases and during aging, where their relationship to disease etiology and early pathogenic events remains incompletely understood. Thus, DAM-like states should be viewed as part of a broader spectrum of context-dependent microglial activation rather than as entities specific to AD [35–39].

DAM resemble aging microglia more than healthy microglia, as both exhibit increased responsiveness and pro-inflammatory phenotype [28, 40]. However, there are distinctive differences seen between DAM and aging microglia with regard to transcriptional and secretion profiles [29, 40]. Recent advances have improved the ability to distinguish microglia across various activation states, including responses to stimuli and changes associated with aging [41]. There is some overlap between aging microglia and DAM, and it has been proposed that aging microglia may eventually transition into DAM, thereby contributing to neuropathology [42]. A critical distinction is the role of ApoE: while aging microglia develop independently of ApoE, DAM formation occurs via an ApoE-dependent pathway, highlighting a molecular mechanism that differentiates AD-specific microglial responses [42]. The transition from homeostatic microglia to DAM in AD is a complex process influenced by a variety of genetic, molecular, and environmental factors, including Aβ, tau, metabolic programming, lipid metabolism, inflammatory microenvironment and signalling pathways, oxidative stress, mitochondrial dysfunction, circadian rhythm dysfunction, cellular interactions, and extracellular matrix alterations [43]. Understanding these factors provides insights into potential therapeutic targets for modulating microglial function in AD.

Recent insights into AD-specific microglial responses have paved the way for novel approaches in early detection and monitoring of the disease. One such promising avenue is the study of microglia-derived EVs, which serve as dynamic indicators of microglial activity and neuroinflammatory processes [44].

## EVs as biomarker carriers

EVs are potent carriers of cellular material, reflecting the physiological state of their cells of origin [45]. They are released by nearly all cell types and can either encapsulate cargo within their lumen or display it on the vesicle surface [46]. Through mechanisms such as receptor-mediated uptake, direct membrane fusion or endocytosis, EVs can transfer their cargo from donor to recipient cells [47]. In this way, EVs function as intercellular messengers, influencing physiological processes by transporting their cargo to both nearby and distant cells [48]. Their ability to carry cellular molecules makes EVs highly attractive candidates as sources of disease-associated biomarkers.

**Fig. 3:**
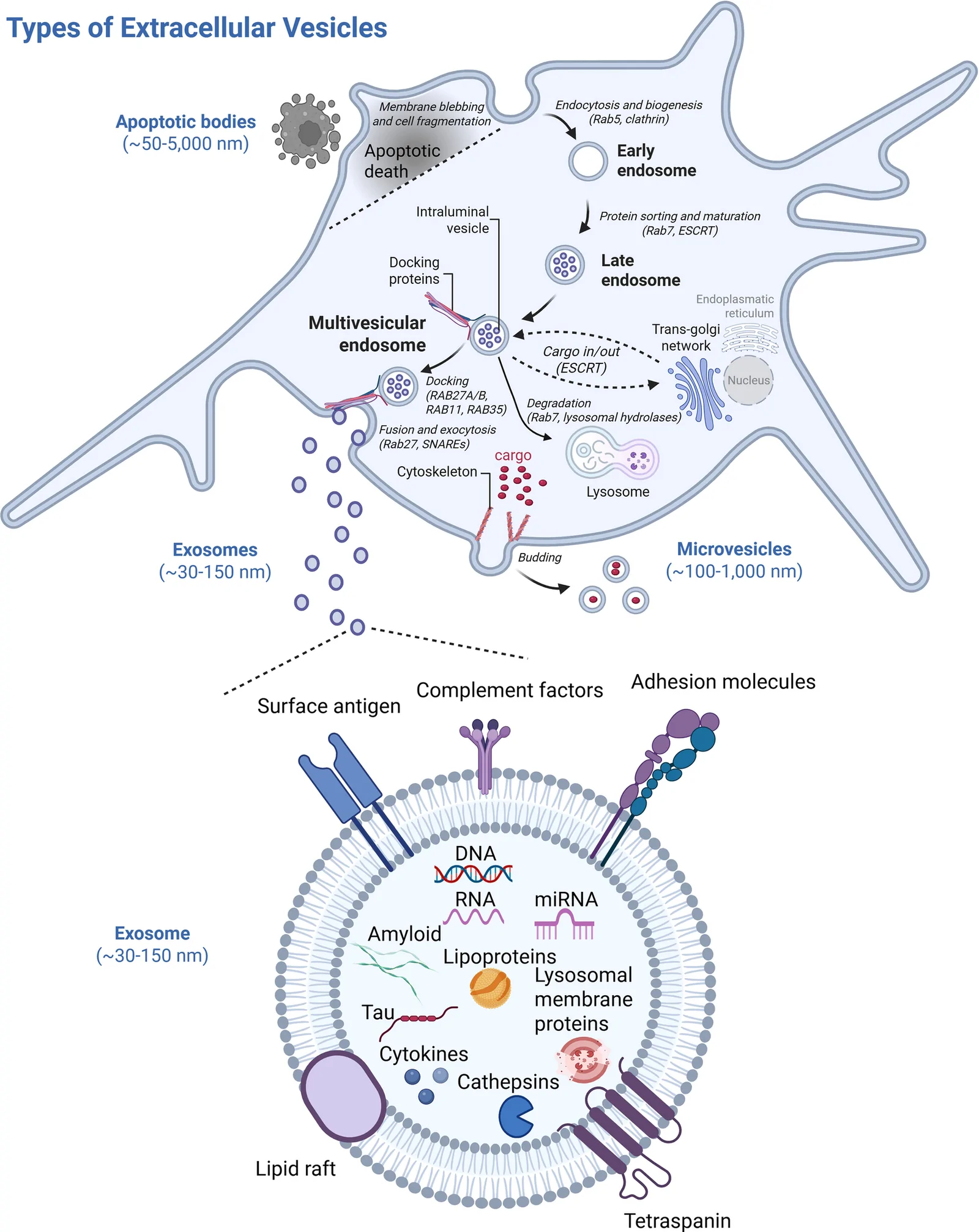
Extracellular vesicle biology. Microglia (both resting and activated) produce three types of extracellular vesicle (EV): exosomes, microvesicles, and apoptotic bodies. Various biomolecules (RNA, DNA, proteins, lipids) can be encapsulated by EVs as well as expressed on their surface (e.g. tetraspanins, CD11b, TMEM119). EV encapsulation protects cargoes from enzymatic degradation (nucleases, proteases), prevents their non-specific interactions with plasma proteins, and enables their efficient delivery to recipient cells via endocytosis or membrane fusion. These EVs can mirror microglial activation which is highly pronounced during the early stages of AD, and carry specific molecular cargoes (e.g., DNA, RNA, miRNA, amyloid, tau, lipoproteins, cathepsins, lysosomal membrane proteins, and cytokines) that can be detected in accessible biological fluids such as CSF and blood

EVs are heterogeneous in size, content, and function, typically ranging from approximately 30 nm to several micrometers, depending on subtype [48]. They are commonly classified into three main types based on biogenesis and physical characteristics: (i) exosomes (~ 30–150 nm), originating from the endosomal system, in which multivesicular bodies transport intraluminal vesicles to the plasma membrane for extracellular release upon membrane fusion; (ii) microvesicles (~ 100–1000 nm), which are formed via outward budding and fission of the plasma membrane, and (iii) apoptotic bodies (~ 50–5000 nm), that arise from membrane blebbing and fragmentation during apoptosis (Fig. 3) [44, 49]. These size ranges partially overlap, and definitive discrimination between EV subtypes typically requires additional molecular and biophysical criteria. EV heterogeneity is complex and also depends on the producing cell type, cargo composition, and physiological conditions [44]. The International Society of Extracellular Vesicles (ISEV) has published guidelines to assist researchers in characterizing EVs and determining the purity of their EV preparation [50]. The Minimal Information for Studies of EVs 2018 (MISEV2018) has advised EV researchers to consider the use of nomenclature that indicates the physical characteristics of EVs such as size (small EVs might be designated, for example, as <100 nm or <200 nm and medium/large EVs might be >200 nm), density, the presence of certain biochemical compositions (such as TMEM+ EVs) or the condition or cell of origin (such as brain-derived EVs) [44, 50].

An important advantage of EVs as biomarker carriers is their ability to shield their cargo in the extracellular space [51]. Unlike free molecules, which are readily degraded by enzymes such as proteases and nucleases, encapsulated cargo remains protected [52]. At the same time, EVs can still be recognized and taken up by other cells circulating in the blood or other fluids [53]. Therefore, EVs have higher stability and longer half-life compared with free molecules [54], which supports more consistent detection and enables longitudinal studies. The encapsulation of microRNAs (miRNAs) within EVs, for example, enables their application as stable biomarkers in neurodegenerative diseases [55]. In contrast, CNS-derived proteins often interact with other components in biological fluids, reducing their detectable fraction; EV-encapsulation prevents such nonspecific interactions [52, 56]. Furthermore, EVs can carry a diverse range of biomolecules (including RNA, DNA, and proteins), broadening the spectrum of potential biomarkers [44, 51, 55, 57].

In summary, the capacity of EVs to protect and deliver diverse molecular cargoes from CNS cells provides a robust and reliable source of measurable biomarkers, offering significant promise for AD and other neurodegenerative disorders.

## Microglia-derived EV content as biomarkers for AD

Microglia-derived EVs represent one of several glia‑derived EV subpopulations in the CNS. Nevertheless, their exact quantitative contribution relative to neuron‑, astrocyte‑ and oligodendrocyte‑derived EVs has not yet been reliably established in humans. In a healthy brain, oligodendrocyte- and astrocyte-derived EVs dominate the glial EV pool, reflecting their high cell density. Microglia constitute roughly 5%–12% of total CNS cells [58, 59], and their homeostatic EV contribution is proportionally low compared to neurons and astrocytes. Current evidence indicates that both the abundance and the cargo of microglial EVs change markedly with the onset and progression of AD [60, 61]. Tracking these changes can provide valuable insights into neuroinflammation and the propagation of neurodegenerative pathology, despite the fact that the CNS-derived EVs account for only about 3%–4% of total circulating plasma EVs [62]. In this context, microglia-derived EVs have emerged as promising early-stage, disease-specific biomarkers for AD [44]. These vesicles mirror microglial activity, which is highly pronounced during the early stages of AD, and carry molecular cargo that can be detected in accessible biological fluids such as CSF and blood [44, 45]. Thus, EVs provide a minimally invasive window into neuroinflammatory processes that occur before clinical symptoms, such as MCI, appear.

Beyond reflecting pathology, EVs can contribute to the propagation of pathologies [63]. For example, microglia may phagocytose Aβ, internalize it into the endosomal–lysosomal system, and either degrade it or route undegraded Aβ or its fragments into intraluminal vesicles that are subsequently released as EVs [63]. Joshi et al. demonstrated that in AD, microglia exposed to insoluble Aβ release EVs enriched in neurotoxic, soluble pre-fibrillar Aβ species in vitro [63]. Lipids within EVs can also destabilize insoluble Aβ aggregates, promoting their conversion into soluble, highly neurotoxic forms that disrupt neuronal membrane integrity and induce calcium influx [63]. In addition to Aβ, brain-derived EVs in AD have been shown to contain seed-competent tau species. A recent study visualized filamentous tau tethered within the lumen of EVs isolated from AD brain tissue, indicating that *bona fide* tau filaments are packaged into EVs in vivo [64]. Complementary work in human iPSC-derived microglia demonstrated that microglia exposed to fibrillar tau internalize it and subsequently release partially digested, seeding-competent tau within EVs, which can induce tau aggregation in recipient neurons [65]. Together, these findings support a role for EVs as vectors for the propagation of both Aβ and tau pathology in AD.

Since nearly all cell types, including CNS cells such as neurons, astrocytes, and microglia, release EVs [46], isolating microglia-specific EVs could enable sensitive and specific early detection of AD-related neuroinflammation. Microglia release EVs under resting conditions, but secretion markedly increases upon activation, for instance through tumor necrosis factor (TNF)-mediated pathways [66]. This increased release is often accompanied by changes in the composition and function of EV cargo (Fig. 3) [47]. For example, TNF and interleukin (IL)-6 are significantly enriched in EVs from activated microglia, whereas IL-5 and IL-1β show increases only in non-encapsulated form, with no corresponding rise in EV cargo [66]. These findings underscore that cytokine levels in EVs do not necessarily mirror those in biofluids and that encapsulation is influenced by activation pathways, environmental context, and disease state. Although further research is needed to define cytokine signatures within microglia-derived EVs across different disease stages, microglia-derived EVs show promise as an early and disease specific biomarker for AD.

In a recent study, microglia-derived small EVs (positive for TMEM119) were isolated from plasma of controls and patients with MCI or AD, and the expression of miRNAs was assessed [67]. Interestingly, the expression of miR-29a-5p could predict MCI (receiver operating characteristic analysis, area under the curve (AUC) = 0.840, indicating good discrimination), while the expression of miR-132-5p and miR-125b-5p could predict AD (AUC = 1.000, indicating perfect separation in this cohort). Notably, the expression of miR-106b-5p (regression coefficient (B) = −0.0171, standard error (SE) = 0.0074, *P* = 0.028) and miR-132-5p (B = −0.0053, SE = 0.0024, *P* = 0.034) showed a significant negative correlation with the temporal cortical thickness [67]. Therefore, the authors concluded that miRNA expression in small EVs could be used as a novel blood-based biomarker for MCI and AD.

Furthermore, it has been shown that the plasma concentration of microglia-derived small EVs (TMEM119-positive; 50–800 nm in diameter, mean 138 nm) is increased in patients with concurrent MCI and frailty (a geriatric syndrome characterized by progressive physical decline and increased vulnerability to endogenous and exogenous stressors, leading to greater dependency and/or mortality) compared with healthy controls and MCI patients without frailty, and that these EVs show high neurotoxic effects on neurons in vitro [68]. This suggests that microglia-derived EV release is associated with the frailty condition and that frailty exacerbates the release of EVs in individuals with MCI.

Interestingly, a multi-omics analysis of small microglial EVs from human AD brain tissue revealed disease-associated signatures, with a significant reduction in the abundance of homeostatic microglia markers P2RY12 and TMEM119, and increased levels of DAM-like markers FTH1 and TREM2, in CD11b-positive EVs from AD brain compared to control cases [61]. The tau abundance was significantly higher in AD brain-derived microglial EVs and that these changes were accompanied by the upregulation of synaptic and neuron-specific proteins in the AD group [61]. Furthermore, levels of free cholesterol were elevated in microglial EVs from the AD brain and this was associated with a proinflammatory lipid profile, endolysosomal dysfunction, and a significant AD-associated decrease in the levels of polyunsaturated lipids containing docosahexaenoic acid (DHA), suggesting a potential defect in acyl-chain remodelling. Additionally, four miRNAs (miR-28-5p, miR-381-3p, miR-651-5p, and miR-188-5p) associated with immune and cellular senescence signaling pathways are significantly upregulated in the AD group [61]. Therefore, the authors concluded that the analysis of microglia-derived EVs has merit for identifying novel EV-associated biomarkers and providing a framework for future larger-scale multi-omics studies on patient-derived cell-type-specific EVs. However, these findings need to be validated in plasma, serum or CSF.

## Measurability of microglia-derived EV biomarkers

EVs are not restricted to local signaling. Their capacity for long-range diffusion enables them to reach distant regions of the CNS [69]. Importantly, EVs can also enter blood circulation and CSF, as they are capable of crossing the blood–brain barrier (BBB), the blood–cerebrospinal fluid barrier and ependymal cells bidirectionally. This happens most likely via combinations of endothelial endocytosis–exocytosis (transcytosis), vesicle release at the abluminal and luminal endothelial surfaces, and bulk flow through glymphatic and meningeal lymphatic pathways [70, 71]. Although the passage of EVs from the brain to the periphery and vice versa is somewhat controversial, with conflicting evidence [70, 71], accumulating data indicate that isolation of CNS-derived EVs from accessible biological fluids such as blood and CSF is possible and feasible [45]. At the same time, a substantial fraction of CNS EVs is likely retained within the brain. Appearance of EVs in peripheral blood can be limited by their local uptake by neighbouring microglia, neurons and astrocytes, interactions with the extracellular matrix, trapping in perivascular spaces, and impaired clearance when BBB integrity is compromised [72, 73]. These processes are expected to depend on EV size and surface composition [72], the expression of endothelial and glial receptors, and the inflammatory and the vascular states of the tissue [73], all of which are altered in AD. Together, these transport and retention mechanisms help explain why only a subset of microglia-derived EVs reaches the circulation and must be considered when interpreting plasma-based EV biomarkers.

EVs offer several practical advantages over the use of whole biological fluids for biomarker discovery. For instance, plasma contains highly abundant proteins that account for ~90% of its total protein content [74, 75]. Similarly, CSF is a complex mixture of molecules from diverse cell types. This makes it challenging to detect low-abundant proteins and it often requires complex purification steps [75]. Although sensitive methods such as enzyme-linked immunosorbent assay (ELISA) or mass spectrometry can be applied, these approaches are relatively time-consuming, technically demanding, and poorly scalable for clinical use [52]. Moreover, enrichment procedures increase sample-to-sample variability, thereby reducing the reliability of free molecules as biomarkers [52].

By contrast, the encapsulation of EV cargo within lipid membranes protects against interaction with abundant proteins and other contaminants, improving measurement accuracy [76]. While EV isolation methods can also introduce variability, reproducibility remains high when standardized protocols are consistently applied [52, 76]. Moreover, EVs carry cell-specific surface markers that allow their origin to be traced back to particular CNS cell types, including microglia (for more details see the following paragraph about specificity) [31].

## Isolation of microglia-derived EVs

Isolating EVs from blood or CSF provides a valuable opportunity to explore their biomarker potential in AD [44]. A variety of EV-specific protein markers are displayed on their surface, including tetraspanins such as cluster of differentiation (CD) 9, CD63, and CD81, heat-shock protein (HSP) 70, and MHC molecules [76]. Notably, CD9 and CD63 also appear within DAM transcriptional modules in an AD mouse model [23], indicating that canonical exosomal markers are themselves part of the DAM gene repertoire, which further supports a mechanistic link between DAM activation states and EV biology. EVs are released into multiple biological fluids, including blood, urine, saliva, and CSF, and can be separated and enriched based on these surface markers [44].

Several methods are available to isolate and concentrate EVs, including ultracentrifugation, density gradient centrifugation, size-exclusion chromatography, microfluidic platforms, chemical precipitation, and biochemical capture. A comprehensive review of these methods has been provided by Veziroglu et al. [48]. While these approaches enable enrichment of EVs, isolating pure populations from biofluids remains challenging. Preparations often contain contaminants such as lipoproteins and non-EV proteins, particularly in blood-derived samples, making additional depletion steps necessary depending on the initial separation methods [77].

Furthermore, Zhang et al. show differences in yield, size, enrichment of EV cargo molecules, and single EV assay by different isolation methods [78], suggesting that the choice of isolation method will have a significant impact on the downstream analysis and protein discovery.

Recent advances in analytical techniques are substantially improving the sensitivity and the resolution of EV-based biomarker studies. High-sensitivity mass spectrometry workflows (e.g., optimized sample preparation, nano-LC, and data-independent acquisition) now allow deep proteomic profiling from low-input EV samples, enabling detection of low-abundance proteins relevant to neurodegeneration [79]. In parallel, nano-flow cytometry and imaging flow cytometry permit single-EV analysis of size and surface markers, making it possible to distinguish EV subpopulations (e.g., neuron- vs microglia-derived vesicles) within complex biofluids [80]. Moreover, multiplex bead-based immunoassays [81] and digital ELISA platforms [82] increase analytical sensitivity for EV-associated proteins and phosphorylated tau species, while targeted sequencing and RT-qPCR panels enhance quantification of EV miRNAs and other RNA cargoes [83]. Together, these methodological advances provide higher sensitivity, better phenotyping of EV subsets, and improved reproducibility, thereby strengthening downstream biomarker discovery and validation.

Comparative evaluations of separation strategies for human serum and CSF have been conducted by Brennan et al. [77] and Muraoka et al. [84], respectively. A recent meta-analysis revealed that studies using CSF for EV isolation, involving non-fasting individuals, employing citrate instead of EDTA (ethylenediaminetetraacetic acid) for blood collection, using thrombin for coagulation factor depletion, isolating EVs with purer methods such as ultracentrifugation and size-exclusion chromatography, not freezing EVs post-isolation, and quantifying miRNA biomarkers instead of proteins, achieved better diagnostic accuracy and less heterogeneity [14].

In a recent preclinical study, a novel method was developed for collecting EVs (exosomes) from the hippocampal interstitial fluid of unanesthetized, freely moving mice using in vivo microdialysis [85]. In this study, proteomics followed by differential expression and pathway analysis showed specific proteins within the EVs that are changed during aging in the APP/PS1 mouse model of AD as compared to wild-type mice [85]. In 9-month-old APP/PS1 mice, the biological pathways uniquely enriched in males were mainly related to metabolic and vascular-associated processes, whereas those uniquely enriched in females were primarily related to metabolic, cellular response, and structural processes.

At the time of release, EVs inherit membrane-associated markers that reflect the identity of their parent cells [47]. For microglia-derived EVs, markers include CD11b, CD68, IBA1, TMEM119, CX3CR1, and pro-inflammatory cytokines, molecules that also indicate microglial activation during neuroinflammation in AD [46, 61, 68, 86]. To the current knowledge, the most specific general microglia marker is TMEM119 [68]. However, homeostatic microglial markers such as TMEM119 and P2RY12 are downregulated in disease states and are not present on all microglial EVs. In addition, more broadly used myeloid markers (IBA1, CD11b, CD68, TREM2) are not exclusive to microglia. See Table 1 for an overview of the markers and their limitations. Interestingly, these markers may be detectable even before the onset of cognitive impairment. However, since EV composition can change under pathological conditions, further research is required to identify stable, disease-relevant markers that reliably confirm the microglial origin. Notably, DAM-like markers such as TREM2 have been identified in CD11b-positive EVs from AD patients [61], highlighting their potential as traceable biomarker carriers. However, TREM2 is not AD-specific: genetic variants in *TREM2* confer risk not only for AD but also for other neurodegenerative disorders, including frontotemporal dementia and Parkinson’s disease, complicating the interpretation of TREM2-related findings as AD-selective biomarkers and underscoring the need to combine TREM2 with additional AD-specific markers and clinical context.

**Table 1:** Microglial markers for extracellular vesicles (EVs)

Class	Marker	Function/State of marker	Change in disease	EV association	Limitations
Protein	TMEM119	Homeostatic microglia	Decreased in disease/microglial activation	Present, decreased in AD	Not present on all microglial EVs
Protein	P2RY12	Homeostatic microglia	Decreased in disease/microglial activation	Present, decreased in AD	May be absent from disease-associated microglial EVs, limiting its use in late-stage disease or highly inflamed tissue
Protein	CX3CR1	Microglia (not exclusive)	Variable	Present	Also expressed by other mononuclear phagocytes (e.g. some peripheral monocytes/macrophages); not strictly microglia-specific, especially in peripheral blood
Protein	IBA1, CD11b, CD68	Pan-microglia/macrophage	Maintained in activation; increased during phagocytosis	Present	Expressed by peripheral macrophages and other myeloid cells; cannot distinguish CNS-resident microglia from infiltrating myeloid cells in peripheral EV samples
Protein	TREM2	Disease/activated	Increased in AD and injury	Increased in AD	Also expressed by other myeloid populations; elevated levels in peripheral EVs may reflect general myeloid activation rather than microglia alone
miRNA	miR-124	Homeostatic/inhibitory	Decreased with microglial activation	Enriched in EVs	Also expressed in neurons and other CNS cells; EV-associated miR-124 detected in blood/CSF is not uniquely microglial; requires combinatorial markers for specificity
mirRNA	miR-146a, miR-155	Pro-inflammatory	Increased with microglial activation	Enriched in EVs	Induced in many immune cells; elevated levels in peripheral EVs are not specific for microglia and may reflect systemic inflammation
Lipid	Cholesterol, proinflammatory lipids	Lipid cargo	Increased in AD and microglial activation	Enriched in EVs	Cholesterol changes occur in EVs from multiple cell types; not unique to microglia-derived EVs; requires context and additional markers for interpretation
Lipid	Pro-inflammatory lipid mediators (e.g. eicosanoids)	Lipid cargo/pro-inflammatory	Increased in AD and microglial activation	Enriched in EVs	Lipid signatures overlap with astrocyte and peripheral immune EVs; specificity for microglia is limited; technical challenges in lipidomics of small EVs

Although standardized protocols for EV isolation and analysis are still required to ensure reproducibility across studies, microglia-derived EV biomarkers hold strong promise for specific isolation due to their distinct surface protein pattern, offering advantages over current non-encapsulated biomarker approaches.

## Sensitivity of microglia-derived EV-based biomarkers

Given that microglia undergo changes even before the onset of MCI, microglia-derived EVs are particularly promising as early and sensitive biomarker sources. Liquid biopsies provide a valuable window into the physiological and pathological state of the CNS. Compared with imaging-based biomarkers, fluid-based biomarkers (derived from CSF or blood) are more widely accessible [87], cost-effective [88], and sensitive to early changes preceding clinical onset [89]. While CSF biomarkers remain the most accurate and predictive for CNS pathology, blood-based biomarkers have advanced significantly and now represent a minimally invasive, practical alternative for longitudinal monitoring [52, 90]. Blood sampling is preferable to lumbar puncture for repeated measurements, enhancing its clinical utility for tracking treatment efficacy and enabling biomarker integration into clinical trials [31, 87].

Recent advances in analytical techniques allow detection and characterization of EV cargoes at the single-vesicle level, enhancing precision in biomarker identification [91]. Moreover, the ability to trace EV cargoes back to specific cell types further increases biomarker sensitivity by linking molecular changes directly to their cellular source.

In summary, EV-derived molecules (particularly those from microglia) offer higher sensitivity as AD biomarkers due to their early presence in disease progression, accessibility through blood samples, and their reduced susceptibility to systemic confounding factors compared with free molecules.

## Specificity of microglia-derived EV-based biomarkers for AD

EVs transport a diverse cargo that includes pathological proteins uniquely associated with AD, offering a disease-specific perspective on CNS processes [45, 51]. A promising strategy for improving specificity in AD involves microglia-derived EVs, particularly those secreted by DAM. DAM possess distinct transcriptional signatures and secrete pro-inflammatory cytokines in patterns that differ from microglia in other neurodegenerative diseases [23]. At the same time, interferon‑responsive, MHC class II-high, lipid‑droplet-accumulating and other microglial subpopulations also emerge during disease onset and progression, and their secretory profiles are likely to shape the overall microglial EV/exosomal milieu. Thus, EV cargoes in AD probably reflect a composite of multiple microglial states rather than DAM alone. Since DAM arise early in AD, their EVs may carry highly specific molecular signatures, such as differentially expressed miRNAs, receptors, cytokines, and proteins that uniquely reflect AD pathology. While most current approaches identify microglia-derived EVs using markers such as CD11b [61] and CD68 [85], future work must establish whether DAM-specific markers can reliably distinguish DAM-derived EVs in biological fluids. The detection of AD-associated markers such as TREM2 in CD11b-positive EVs supports this possibility, as does the observation that levels of TREM2, CD14 and CD68 vary according to the phenotype of the cell, suggesting cell-state specificity [61, 92, 93]. It is important to keep in mind that rather than a uniform entity, DAM likely represent a spectrum of states shaped by disease context, progression, environment, and genetics [94]. In addition, transcriptional and metabolic differences between homeostatic microglia and DAM from rodents and humans as well as from males and females have been shown [95]. Furthermore, DAM-specific markers of AD have been identified in the 5×FAD mouse model, including increased gene expression of *Trem2, Axl, Cst7, Ctsl, Lpl, Cd9, Csf1, Ccl6, Itgax (Cd11c), Clec7a, Lilrb4* and *Timp2* [23]. However, microglia do not express many cell-specific proteins in a stable manner and they share significant expression profiles with peripheral macrophages [96]. Further, once the microglia-derived EVs enter the circulation, they become part of a heterogeneous mixture of extracellular particles, including EVs from various cell types and non-vesicular entities such as lipoproteins and soluble proteins [97]. This heterogeneity complicates the attribution of EVs to specific tissue origins, as most methods for preparing EVs from biofluids capture a broad range of particles with low specificity [97]. This represents a significant hurdle for establishing the relevance of EV-based diagnostics to disease and clinical translation. Accurately tracing the tissue origins of EVs depends on the presence of specific surface-associated markers that can be targeted using affinity-based methods [86]. However, suitable markers are often limited across different tissues [97], highlighting the need for rigorous characterization and validation when using tissue-specific EVs as biomarkers.

Beyond studies that specifically enrich for microglia-derived EVs, several investigations have characterized mixed or broadly defined EV populations in AD using CSF, plasma or brain tissue. For example, Oosterlynck et al. analysed EVs from brain-derived fluid of AD patients and controls and reported alterations in immune- and microglia-associated pathways in AD-derived EVs, indicating that disease-related changes in microglial signalling are detectable even when EV preparations are not explicitly microglia-enriched [98]. This supports the notion that microglia-derived EVs likely remain a numerical minority within the total CNS EV pool, but that their relative contribution to disease-relevant cargo and signalling is amplified during AD onset and progression.

Although the heterogeneity of microglia makes EV identification challenging, it also represents an important opportunity to develop biomarkers specific to disease states [96]. To confirm the specificity of DAM-associated biomarkers, their protein expression profiles must be investigated throughout disease progression. Interestingly, in a preclinical stroke model, EVs (type not specified) released from microglia reflect BBB damage and carry fibrinogen as cargo [99]. Importantly, this cargo can be detected in circulating plasma EVs, supporting their utility as peripheral biomarkers of microglia activity [99]. ScRNA-seq and snRNA-seq studies in various AD mouse models have revealed variability in DAM transcriptional profiles depending on the model used [30]. Similar variability has been observed in patient CSF samples, suggesting that scRNA-seq/snRNA-seq clustering can influence transcriptional patterns. Moreover, Chen et al. reported distinct differences between DAM-like gene expression in mice and that in patients with AD [30], indicating that murine DAM clusters do not map one-to-one onto human AD microglial states and underscoring that DAM-based biomarker candidates derived from mouse models require careful validation in human tissues and biofluids. The DAM state was originally defined in 5×FAD mouse models, where microglia adopt a two‑step TREM2-APOE-dependent transcriptional program characterized by upregulation of genes such as *Apoe*, *Trem2*, *Tyrobp*, *Spp1*, *Cst7*, *Lpl* and *Itgax* [23]. Single‑cell and single‑nucleus transcriptomic studies of human AD cortex have identified microglial clusters with partial overlap with this DAM module, including increased expression of *APOE*, *TREM2*, *TYROBP*, *SPP1* and related genes, and these are often referred to as ‘DAM‑like’ [24, 100, 101]. However, the human AD microglial landscape is more heterogeneous and includes additional inflammatory, interferon‑responsive and antigen‑presenting states, and not all canonical murine DAM markers are consistently induced in human tissue [24, 30]. Thus, DAM‑like signatures in human brain should be considered as related but non‑identical counterparts of murine DAM, and extrapolations from mouse to human require careful validation.

In a few meta-analyses, specific biomarkers in EVs (exosomes) from plasma and serum are reported in the context of AD, including Aβ1–42, P-T181-tau, P-S396-tau, and T-tau, as well as molecules related to additional risk factors that are involved in neuroinflammation (C1q), metabolism disorder (P-S312-IRS-1), neurotrophic deficiency (HGF), vascular injury (VEGF-D), autophagy-lysosomal system dysfunction (cathepsin D), transcription-related factors (REST) and miRNAs (e.g., miR-132) [102–104]. Very recently, a diagnostic accuracy meta-analysis and meta-regression was performed focusing on studies attempting to differentiate between prodromal (MCI) or various clinically established dementias (including AD) from one another, using biomarkers derived from general EVs (from studies employing techniques to isolate EVs without further immunocapture of cell-specific EVs) or speculative CNS-enriched EVs (from studies using enrichment antibodies to immunocapture cell-specific EVs from the CNS) [14]. The term ‘speculative’ is used, since the CNS origin of EVs remains uncertain, as commonly used markers lack specificity and may not reliably associate with EVs. Furthermore, the extensive uptake and recycling of EVs across many cell types raises questions about whether their cargo can accurately reflect CNS-derived signals. Yet, this meta-analysis reveals that biomarkers derived from general (non-CNS enriched) EVs offer better diagnostic accuracy, exhibit less heterogeneity, and demonstrate substantially lower publication bias compared to speculative CNS-enriched EVs [14]. Across all studies using general EVs, the pooled diagnostic performance for distinguishing AD from negative controls was moderate, with an AUC of 0.84 (partial AUC 0.82), sensitivity 0.83 (95% CI 0.77–0.88), and specificity 0.79 (95% CI 0.73–0.84) [14]. By contrast, diagnostic accuracy for differentiating AD from MCI (pooled AUC 0.70; sensitivity 0.68; specificity 0.78), frontal temporal dementia (AUC 0.68; sensitivity 0.70; specificity 0.71), or dementia with Lewy body/Parkinson’s disease dementia (AUC 0.83; sensitivity 0.74; specificity 0.89) was only modest [14]. Notably, the comparison between AD and VCID (vascular cognitive impairment and dementia) achieved the highest diagnostic accuracy, with general EV biomarkers yielding a pooled AUC of 0.97 (partial AUC 0.95), sensitivity 0.95 (95% CI 0.82–0.99), and specificity 0.96 (95% CI 0.69–1.00) [14].

Taken together, current data suggest that EV-derived biomarkers have the potential to distinguish AD from normal ageing, but the diagnostic accuracy reported to date is only moderate and remains insufficient for routine clinical application, particularly when differentiating AD from other dementia syndromes. Whether DAM-derived EVs offer additional specificity needs to be investigated.

## Conclusions and future perspectives

This review provides the current status of microglia-derived EV content as biomarkers for early detection of AD. Because they may arise before overt pathology and can be accessed with minimal invasiveness, microglia‑derived EVs represent a promising avenue for future biomarker development. Nevertheless, their disease specificity and diagnostic sensitivity for AD remain incompletely defined. Beyond early diagnosis, microglia-derived EVs may also support longitudinal disease monitoring and treatment evaluation.

Building on this potential, a critical next step is to move beyond general EV signatures and explore DAM-specific EV profiles, particularly during early disease stages of AD. Candidate biomarkers include pro-inflammatory cytokines (TNF-α, IL-1β, IL-6), anti-inflammatory cytokines (IL-10, transforming growth factor β), miRNAs, and additional molecules such as matrix metalloproteinases, HSPs, and complement proteins. These candidates must be validated for their correlation with DAM-derived EVs and their ability to distinguish early-stage AD from normal aging or other dementias.

There are, however, still considerable obstacles in the adoption of DAM-specific EV-based biomarker strategies for AD. The necessary technology to measure EVs is not commonly accessible, and achieving consistent measurements across different centers requires standardized protocols and technical expertise. Cell specificity is a persistent issue with any blood-based EV assay. To attain specificity, it is likely necessary to implement EV biomarker profiles that utilize multiple surface proteins along with cargo analysis. If EVs display microglia markers that can also be found in peripheral cells, but contain CNS-derived cargoes (such as brain specific proteins), it is more likely that the content of this EV originates from the brain. Despite these difficulties, we believe the advantages of EVs surpass their drawbacks. These advantages include the ease of measuring EVs in blood compared to CSF-based assays, the long-term stability of EVs when stored at −80 °C, and their ability to reflect CNS-specific inflammatory signaling.

Ultimately, the development of a DAM-derived EV biomarker panel requires rigorous validation in large patient cohorts. Establishing their sensitivity, specificity, and predictive value will be critical before such biomarkers can be translated into universal clinical use. Further research is also needed to define early AD-specific protein expression profiles of DAM in patients and to map their progression from initial appearance to full disease manifestation. Defining a robust set of DAM-derived EV biomarkers could provide a transformative tool for earlier diagnosis, more precise monitoring, and earlier intervention in AD.

## Data Availability

No datasets were generated or analysed during the current study.

## Abbreviations

AD: Alzheimer’s disease
BBB: Blood-brain barrier
CD: Cluster of differentia
CNS: Central nervous system
CSF: Cerebral spinal fluid
DAM: Disease-associated microglia
ELISA: Enzyme-linked immunosorbent assay
EVs: Extracellular vesicles
HSP: Heat-shock protein
IL: Interleukin
MCI: Mild cognitive impairment
MGnD: Microglial Neurodegenerative phenotpye
MHC: Major histocompatibility complex
PET: Positron emission tomography
scRNA-seq: Single-cell RNA sequencing
snRNA-seq: Single-nuclei RNA sequencing
TNF: Tumor necrosis factor
TREM2: Triggering receptor expressed on myeloid cells 2
